# HQRNN-FD: A Hybrid Quantum Recurrent Neural Network for Fraud Detection

**DOI:** 10.3390/e27090906

**Published:** 2025-08-27

**Authors:** Yao-Chong Li, Yi-Fan Zhang, Rui-Qing Xu, Ri-Gui Zhou, Yi-Lin Dong

**Affiliations:** 1 College of Information Engineering, Shanghai Maritime University, 1550 Haigang Avenue, Pudong New Area, Shanghai 201306, China; ycli@shmtu.edu.cn (Y.-C.L.); rgzhou@shmtu.edu.cn (R.-G.Z.); yldong@shmtu.edu.cn (Y.-L.D.); 2 Research Center of Intelligent Information Processing and Quantum Intelligent Computing, Shanghai Maritime University, 1550 Haigang Avenue, Pudong New Area, Shanghai 201306, China; 3 Faculty of Intelligence Technology, Shanghai Institute of Technology, 100 Haiquan Road, Fengxian District, Shanghai 201418, China; xrq@sit.edu.cn

**Keywords:** quantum computing, quantum finance, quantum machine learning, variational quantum circuits, hybrid quantum–classical models, financial fraud detection

## Abstract

Detecting financial fraud is a critical aspect of modern intelligent financial systems. Despite the advances brought by deep learning in predictive accuracy, challenges persist—particularly in capturing complex, high-dimensional nonlinear features. This study introduces a novel hybrid quantum recurrent neural network for fraud detection (HQRNN-FD). The model utilizes variational quantum circuits (VQCs) incorporating angle encoding, data reuploading, and hierarchical entanglement to project transaction features into quantum state spaces, thereby facilitating quantum-enhanced feature extraction. For sequential analysis, the model integrates a recurrent neural network (RNN) with a self-attention mechanism to effectively capture temporal dependencies and uncover latent fraudulent patterns. To mitigate class imbalance, the synthetic minority over-sampling technique (SMOTE) is employed during preprocessing, enhancing both class representation and model generalizability. Experimental evaluations reveal that HQRNN-FD attains an accuracy of 0.972 on publicly available fraud detection datasets, outperforming conventional models by 2.4%. In addition, the framework exhibits robustness against quantum noise and improved predictive performance with increasing qubit numbers, validating its efficacy and scalability for imbalanced financial classification tasks.

## 1. Introduction

Financial fraud, often manifested through anomalous or unauthorized transaction activity, presents a serious risk to individual fund security and the overall integrity of financial systems. The growing pace of digital transformation has given rise to increasingly sophisticated and varied fraudulent schemes. By 2023, global financial fraud was estimated to have caused losses exceeding USD 300 billion, with an annual growth rate of approximately 15% [1]. This escalating trend not only undermines the operational effectiveness of financial institutions but also diminishes user confidence and places considerable strain on existing regulatory mechanisms within the fintech sector [2].

To counter the increasing complexity of fraud, deep learning-based approaches have been widely employed for fraud detection tasks. Recurrent neural networks (RNNs) and their enhanced variants—such as long short-term memory and gated recurrent units—have proven effective due to their capability to capture long-range temporal dependencies in financial transaction data [3,4]. Despite these advancements, the deployment of deep learning models in practical settings continues to face three key challenges:

- RNN-based architectures are prone to gradient vanishing or explosion when handling very long transaction sequences, limiting their effectiveness in modeling complex behavioral patterns [5];
- Traditional deep learning models often struggle to capture nonlinear relationships accurately in high-dimensional and highly dynamic transaction data [6];
- The dynamic and adversarial characteristics of fraudulent activities present major challenges for conventional deep learning models, as these models struggle to generalize to novel fraud patterns that are absent from the training dataset.

Quantum computing has emerged as a promising avenue for financial fraud detection due to its ability to perform nonlinear transformations in high-dimensional feature spaces [7]. Unlike conventional machine learning methods, quantum computing leverages quantum superposition and entanglement to uncover salient fraud patterns within exponentially large feature representations, thereby enhancing model expressiveness in complex financial contexts [8,9]. However, the current generation of quantum hardware continues to face notable limitations in computational accuracy, qubit scalability, and measurement stability, rendering the large-scale deployment of purely quantum models impractical in the near term [10].

In response to persistent challenges in high-dimensional feature representation and class imbalance, hybrid quantum–classical models have gained traction. These architectures integrate variational quantum circuits (VQCs) with classical deep learning frameworks, harnessing the representational capacity of quantum systems while ensuring compatibility with noisy intermediate-scale quantum (NISQ) hardware. Following this direction, the present study introduces hybrid quantum recurrent neural network for fraud detection (HQRNN-FD), a hybrid quantum–classical model that synergistically combines quantum encoding, temporal sequence modeling, and attention mechanisms to enhance the detection of anomalous transaction behaviors in financial datasets. The primary contributions of this work are summarized as follows:

- End-to-end quantum–classical hybrid sequence modeling framework: This work introduces a unified architecture that integrates VQCs and RNNs within a shared computational graph. This design enables the simultaneous optimization of quantum gate parameters and neural network weights, supporting dynamic sequence modeling enriched by quantum-augmented feature representations.
- Hierarchical entanglement with angle encoding: A multi-layer entanglement structure combined with parameterized rotation gates is implemented in the quantum encoding module. Transactional features are embedded into high-dimensional quantum state spaces using angle encoding and data reuploading, enhancing the model’s ability to capture nonlinear dependencies and long-range behavioral patterns.
- Self-attention integration for improved pattern discrimination: Self-attention mechanisms are incorporated both within RNN cells and at the discriminative output layer. This two-tier attention structure strengthens temporal feature extraction and refines decision making, enabling the model to focus on salient behavioral signals—particularly valuable in class-imbalanced scenarios.
- Synthetic minority oversampling technique (SMOTE)-based strategy for class imbalance mitigation: The SMOTE is employed during preprocessing to augment the representation of minority-class (fraudulent) samples. This preprocessing step improves class balance and supports better generalization to novel fraud instances.
- Extensive empirical evaluation and robustness testing: The proposed HQRNN-FD model is rigorously evaluated through comparative experiments against classical and quantum baselines, consistently achieving superior accuracy and F1-scores. Additional tests under varying qubit counts and quantum noise conditions validate the model’s scalability and its practical viability on current quantum hardware.

The remainder of this paper is structured as follows. Section 2 provides a review of related work in financial fraud detection and quantum computing. Section 3 details the architectural design and key components of the proposed HQRNN-FD model. Section 4 outlines the datasets used and the preprocessing procedures applied. Section 5 describes the experimental setup, evaluation metrics, and comparative analysis of results. Finally, Section 6 concludes the study and outlines directions for future research.

## 2. Preliminary Information

### 2.1. Financial Fraud Detection

Financial fraud, a critical component of financial risk, has long been a primary concern in both academic research and industry applications. Traditional detection methods, largely based on rule-driven systems and statistical modeling, have become increasingly inadequate in the face of growing transaction volumes, rising frequency, and the complexity of financial behaviors. These conventional approaches often suffer from delayed detection, limited predictive accuracy, and poor adaptability to evolving fraud patterns [11,12].

Recent advancements in machine learning have significantly improved performance in fraud detection tasks. For example, Xie et al. [13] introduced a transaction behavior representation learning framework that leverages a spatiotemporal joint model to extract fine-gained behavioral features, thereby enhancing the identification of latent fraudulent activities. To address issues related to class imbalance and the scarcity of labeled fraud instances, Zhu et al. [14] proposed adaptive optimization methods that combine feature selection with data sampling strategies, effectively reducing detection bias and improving model robustness.

Further progress has been made through ensemble approaches. Khalid et al. [15] developed an ensemble machine learning framework to improve detection stability, while Yang et al. [16] proposed a mixture-of-experts model combined with deep learning–based data rebalancing to enhance credit fraud detection.

Despite these advancements, existing models often face limitations in terms of generalizability and real-time performance, especially in high-dimensional transaction environments. As a result, there is growing interest in quantum machine learning (QML) as a potential solution to these challenges, with emerging research exploring its applicability to financial fraud detection.

### 2.2. Quantum Computing

Quantum computing is an emerging computational paradigm that exploits core principles of quantum mechanics—namely superposition, entanglement, and interference—to encode and manipulate information. Unlike classical bits, the fundamental unit of quantum computation, the qubit, can exist in a superposition of states, allowing processing capabilities that scale exponentially with the number of qubits.

Unlike classical computing, which relies on irreversible logic gates, quantum computing performs operations using unitary quantum gates—such as Hadamard, Pauli-X, and CNOT—that maintain coherence and reversibility throughout computation [17]. Quantum entanglement further enables intricate correlations across qubits, facilitating high-dimensional state encoding and complex multi-variable interactions.

Egger et al. [18] noted that quantum computing applications have extended well beyond physical simulations to include cryptography, drug discovery, combinatorial optimization, and financial modeling. Orús et al. [19] identified three key areas where quantum computing offers a potential advantage in finance: combinatorial optimization (e.g., portfolio selection), Monte Carlo simulation speedups (e.g., risk modeling), and QML (e.g., fraud detection and time-series prediction).

Although current quantum hardware remains within the NISQ era, research and development efforts have already demonstrated its potential in various financial applications—including customer segmentation, credit risk assessment, and fraud detection—driven by growing academic and industrial collaborations [20].

### 2.3. Quantum Computing for Finance

Quantum computing has recently emerged as a promising frontier in financial computation due to its potential to address high-dimensional optimization problems and complex constraint modeling. Many financial tasks, including asset portfolio allocation, derivative pricing, and risk exposure assessment, are characterized by large feature spaces, nonlinear dependencies, and combinatorial constraints. These challenges often fall into the category of NP-hard problems, making them suitable candidates for quantum-based solutions. For instance, Shunza et al. [21] demonstrated how quantum circuits can be effectively applied to discrete portfolio optimization problems under practical constraints.

Orús et al. [19] highlighted that quantum methods, such as quantum annealing and amplitude estimation, can accelerate processes, such as asset selection, covariance estimation, and Monte Carlo simulation, thereby offering significant improvements in financial risk modeling. Egger et al. [18] presented a comprehensive review of quantum computing applications in finance, covering use cases, such as value-at-risk (VaR) estimation [22], portfolio rebalancing, and strategy generation. They also emphasized that hybrid quantum–classical models [23] provide a viable solution under the constraints of current NISQ hardware. Building on this, Bhasin et al. [24] proposed advanced QML algorithms for portfolio optimization, demonstrating enhanced scalability and robustness in NISQ settings.

Srivastava et al. [25] applied quantum regression techniques and superposition-based feature encoding to stock market prediction tasks, resulting in improved accuracy and greater sensitivity to high-frequency nonlinear patterns compared with conventional neural models. In real-world applications, Gómez et al. [26] surveyed quantum computational finance approaches including applications in derivatives pricing, VaR, and potential uses in customer segmentation, credit scoring, and fraud detection.

In summary, quantum computing not only offers theoretical advantages in reducing computational complexity but also shows growing potential for delivering tangible improvements in areas such as risk management, predictive analytics, and financial optimization.

### 2.4. Quantum Machine Learning for Fraud Detection

QML, which combines the parallel processing capabilities of quantum computing with the flexibility of classical learning frameworks, has emerged as a promising approach for financial fraud detection. Compared with traditional models, QML methods exhibit improved capacity to learn fine-grained and heterogeneous features from transactional data, even with limited training samples. This advantage is largely attributed to quantum feature encoding and the use of VQCs, which enhance both discriminative ability and generalization, particularly in class-imbalanced settings [17].

Emmanoulopoulos and Dimoska [8] developed a quantum time-series model that integrates quantum encoding into sequential architectures, significantly improving the modeling of complex temporal dependencies in fraud detection tasks. Similarly, Innan et al. [11] explored a range of QML models and identified quantum graph neural networks as particularly effective in capturing the relational structure of transaction networks. In another study, Gandhudi et al. [27] proposed explainable hybrid quantum neural networks (QNNs) to assess the impact of external social signals (such as tweets) on stock prices, demonstrating the relevance of interpretable QML models in both anomaly detection and financial forecasting.

To address ongoing limitations related to quantum noise, model adaptability, and feature representation, this work proposes HQRNN-FD—a hybrid quantum–classical model that integrates an RNN backbone with a self-attention mechanism and quantum angle encoding. The architecture also employs data reuploading, allowing classical features to interact repeatedly with layered VQCs, thereby enabling richer sequential representations [28].

Thakkar et al. [29] recently demonstrated the practical effectiveness of QML in financial forecasting, highlighting the viability of hybrid architectures in real-world environments [30]. Although HQRNN-FD does not explicitly incorporate robustness regularization, it is evaluated under simulated quantum noise conditions that reflect current NISQ hardware. The experimental results show that HQRNN-FD consistently outperforms classical deep learning and existing QML baselines across multiple imbalanced fraud detection datasets, achieving superior performance in terms of accuracy, recall, and F1-score. Furthermore, the model demonstrates strong resilience to quantum noise, indicating its suitability for real-time anomaly detection in high-risk financial transaction scenarios [31].

## 3. Methods

### 3.1. Design of Variational Quantum Circuit Architecture

To improve the modeling of nonlinear structures and temporal dependencies in complex financial transaction data, this study incorporates VQCs as a quantum feature extraction component within a hybrid deep learning framework. Leveraging the principles of quantum superposition and entanglement, the VQC module projects classical transaction features into a high-dimensional Hilbert space. This transformation facilitates the detection of subtle anomalous behaviors while enabling expressive nonlinear feature expansion with relatively few trainable parameters.

**Figure 1:**
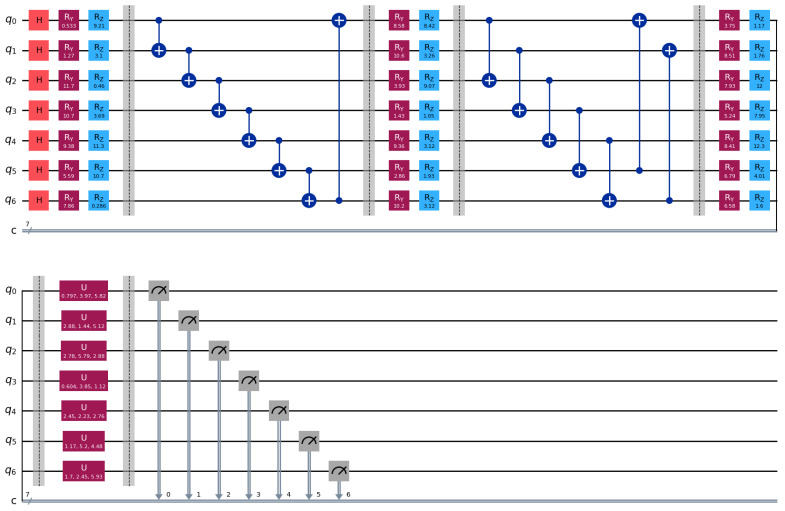
Architecture of the variational quantum circuit. Red blocks denote the Hadamard gate and $R_{y}$ rotation gates, blue blocks represent the $R_{z}$ rotation gates, purple blocks indicate parameterized unitary (*U*) gates, and gray blocks correspond to measurement operations. Blue circles connected with lines represent controlled operations (CNOT gates).

Figure 1 depicts the full architecture of the VQC module. The process begins with the initialization of classical input features as quantum states, followed by nonlinear angle encoding. These encoded states are then processed through multiple layers of entanglement and parameterized rotation gates. Finally, quantum state information is converted back into classical tensors through measurement operations. This quantum module is embedded within a unified hybrid architecture, allowing for end-to-end joint training with RNNs and attention mechanisms. The following sections provide a detailed description of the complete model architecture.

#### 3.1.1. Quantum State Encoding

QNNs traditionally employ amplitude-based or basis encoding schemes to project classical data into the quantum Hilbert space. While these approaches can reduce input dimensionality in certain contexts, they often necessitate deeper circuit structures and are generally limited in their ability to capture complex nonlinear discriminative patterns. Moreover, directly embedding raw input values into quantum amplitudes or measurement outcomes can lead to numerical instability and heightened sensitivity to quantum noise, particularly when handling inputs with large dynamic ranges or outliers.

From a theoretical perspective, variational quantum circuits are particularly well-suited to fraud detection tasks involving nonlinear, high-dimensional, and sparse data. By leveraging quantum superposition, each qubit can encode multiple states simultaneously, enabling an implicit feature space whose dimension grows exponentially with qubit count. Entanglement further allows the model to capture complex inter-feature dependencies that classical linear transformations cannot easily model. This capability is advantageous for financial fraud detection, where fraudulent patterns may be embedded as subtle, nonlinear correlations across heterogeneous transactional attributes.

The adoption of variational quantum circuits (VQCs) in this study is theoretically motivated by their ability to map classical data into high-dimensional Hilbert spaces whose representational capacity, in the ideal setting, scales exponentially with the number of qubits. Even under current NISQ-era constraints, this property allows VQCs to capture intricate inter-feature correlations that are challenging for purely classical architectures to model. For financial fraud detection—where the feature space is often nonlinear, high-dimensional, and sparse—VQCs can leverage quantum entanglement to represent complex decision boundaries beyond the reach of conventional models. Additionally, the inherent stochasticity of quantum measurement can act as a form of regularization, potentially mitigating overfitting in sparse-data scenarios. These combined properties make VQCs particularly well-suited for learning discriminative patterns in noisy and imbalanced financial datasets.

To mitigate these issues, this study introduces a dual-rotation angle encoding strategy that maps transaction features into both amplitude and phase channels through nonlinear transformations. This method employs parameterized $R_{y}$ and $R_{z}$ gates to enhance the expressive capacity of quantum states.

Prior to encoding, Hadamard gates are applied to all qubits to initialize the system in a uniform superposition state. This operation distributes quantum amplitude uniformly across the $2^{N}$-dimensional Hilbert space, thereby maximizing initial entropy and ensuring high representational capacity for subsequent encoding:

(1) $$|\psi_{0}\rangle =H^{⊗N}{|0\rangle }^{⊗N}$$

where *N* represents the number of qubits, equivalent to the dimensionality of the classical feature vector, and $H^{⊗N}$ denotes the application of Hadamard gates to all qubits, producing the uniform superposition state.

**Figure 2:**
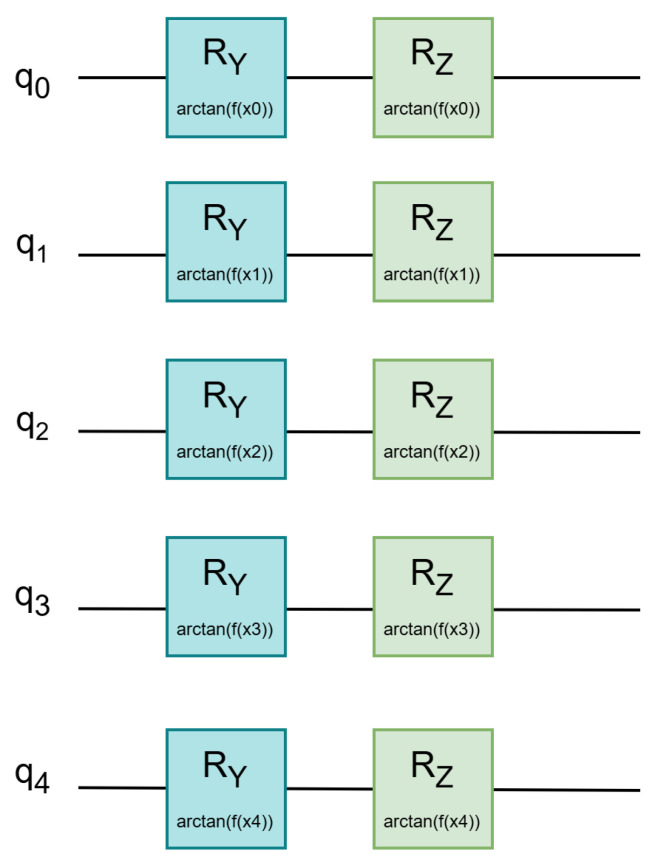
Quantum circuit structure with parameterized rotation gates for angle-based encoding.

Following initialization, classical features are embedded into the quantum system using the proposed dual-angle rotation scheme. Each qubit is subjected to two sequential parameterized rotations; an $R_{y}$ gate encodes the normalized feature value along the *Y*-axis, modulating the state amplitude, while an $R_{z}$ gate encodes the squared value of the input along the *Z*-axis, introducing phase modulation. As shown in Figure 2, this structure allows for joint amplitude–phase encoding, thereby capturing richer and more nuanced feature representations within the quantum state space.

To enhance nonlinearity and ensure bounded rotation parameters, we apply the arctangent function to transform classical input features into rotation angles. This transformation ensures that the mapped angles remain within the valid physical interval and introduces nonlinear characteristics beneficial for encoding financial transaction patterns. To ensure that the rotation angles remain within the physically valid interval ($-\frac{\pi }{2},\frac{\pi }{2}$), all input features are standardized prior to encoding. This preprocessing step maintains numerical stability during angle mapping and prevents extreme values from causing invalid quantum operations. Following this standardization, each feature is embedded into a quantum state using the dual-angle rotation scheme. The resulting state of the *i*-th qubit is expressed as:

(2) $$|x_{i}\rangle =R_{z}\left(\phi_{i}\right)R_{y}\left(\theta_{i}\right)|0\rangle$$

where $R_{y}\left(\theta_{i}\right)$ represents the amplitude rotation about the Y-axis, and $R_{z}\left(\phi_{i}\right)$ encodes phase information through a Z-axis rotation. The rotation angles are defined as $\theta_{i}=\arctan\left(x_{i}\right)$ for amplitude modulation and $\phi_{i}=\arctan\left(x_{i}^{2}\right)$ for phase modulation, allowing the nonlinear encoding of both raw and squared feature values into the quantum state. This added sentence explicitly states the nonlinear transformations applied during encoding.

This dual-angle encoding scheme provides a richer representation of each transaction’s features by capturing information in both amplitude and phase. Specifically, amplitude modulation encodes the raw input values while phase modulation incorporates nonlinear variations through squared inputs. This joint embedding strategy enables the quantum circuit to model complex nonlinear dependencies inherent in financial transactions. Such a capability is particularly advantageous for fraud detection, where fraudulent behaviors often manifest as subtle deviations from normal patterns. By enriching the encoded information in both quantum channels, the model is better equipped to identify rare but significant anomalies.

#### 3.1.2. Data Reuploading Strategy

To further enhance the representational capacity of quantum state encoding, this study adopts a data reuploading mechanism. Rather than embedding classical features only once, the normalized input features are re-encoded and injected into multiple layers of the quantum circuit. Specifically, after the initial rotation and entanglement layers, the same set of classical features is reintroduced into the quantum circuit using the same parameterized rotation gates. This repeated injection allows the circuit to see the input multiple times across depths, significantly enriching its expressive power without increasing the number of qubits.

This technique addresses the limited expressiveness of shallow circuits and supports dynamic information interaction across circuit depths. Each reuploading unit consists of a repeated application of the dual-angle encoding gates $R_{y}\left(\theta_{i}\right)$ and $R_{z}\left(\phi_{i}\right)$ to each qubit, placed between successive entanglement layers, allowing the quantum state to evolve under multiple views of the same input. This process is formally expressed as:

(3) $$|x_{i}^{\left(l\right)}\rangle =R_{z}\left(\phi_{i}\right)R_{y}\left(\theta_{i}\right)|\psi_{i}^{\left(l\right)}\rangle$$

where $|\psi_{i}^{\left(l\right)}\rangle$ denotes the quantum state of the *i*-th qubit at the *l*-th layer, and $\theta_{i}=\arctan\left(x_{i}\right)$, $\phi_{i}=\arctan\left(x_{i}^{2}\right)$ are the nonlinear rotation parameters introduced in the encoding phase.

This iterative embedding mechanism enhances the quantum circuit’s ability to capture nonlinear, hierarchical dependencies using a limited number of qubits. By re-encoding the same input in later layers, the model effectively simulates deeper feature interactions. As illustrated in Figure 1, data reuploading is interleaved with parameterized rotation and entanglement blocks, forming a layered quantum architecture that facilitates deeper quantum-temporal feature modeling for downstream fraud classification tasks.

#### 3.1.3. Parameterized Quantum Circuits

In the context of financial fraud detection, anomalous transaction patterns often appear as abrupt bursts—such as unusually large transactions conducted through multiple terminals within short timeframes, or coordinated irregular activities across linked accounts. Capturing such dynamic and spatially distributed behaviors poses a challenge for shallow or rigidly structured quantum circuits, which typically lack sufficient capacity to model inter-qubit correlations.

Furthermore, many conventional quantum encoding schemes are static and non-trainable, limiting their ability to adapt to specific task objectives. To address these challenges, this study introduces a parameterized quantum circuit (PQC) architecture that incorporates hierarchical entanglement layers and universal single-qubit rotation gates. The design consists of three key components.

First, a local entanglement layer is used to establish short-range correlations by connecting adjacent qubits through a closed-loop CNOT gate structure. For a register of qubits $\left\{q_{0},q_{1},\ldots ,q_{N-1}\right\}$, the local entanglement operation is defined as:

(4) $$\mathrm{CNOT}\left(q_{i},q_{(i+1)\;\mathrm{mod}\;N}\right),\;i\in \left\{0,1,\ldots ,N-1\right\}$$

Second, to capture long-range dependencies, a global entanglement layer introduces CNOT operations between alternating qubit pairs, enabling non-local interactions. This configuration is expressed as:

(5) $$U_{\mathrm{ent}}^{\left(2\right)}=\prod_{j=1}^{N/2}\mathrm{CNOT}\left(q_{2j-1},q_{2j}\right)$$

Third, each qubit is equipped with a universal parameterized gate $U_{3}\left(\alpha ,\beta ,\gamma \right)$, which comprises a sequence of three single-qubit rotation gates. This unit serves as the trainable component of the PQC and governs the evolution of quantum states:

(6) $$U_{3}\left(\alpha ,\beta ,\gamma \right)=R_{z}\left(\gamma \right)R_{y}\left(\beta \right)R_{z}\left(\alpha \right)=\left[\begin{array}{cc} e^{-i(\alpha +\gamma )/2}\cos\left(\beta /2\right) & -e^{-i(\alpha -\gamma )/2}\sin\left(\beta /2\right)\\ e^{i(\alpha -\gamma )/2}\sin\left(\beta /2\right) & e^{i(\alpha +\gamma )/2}\cos\left(\beta /2\right) \end{array}\right]$$

In this formulation, α, β, and γ are trainable parameters that define rotations along arbitrary axes on the Bloch sphere. These parameters provide fine-grained control over the quantum state evolution, significantly enhancing the circuit’s capacity to model complex, nonlinear patterns in transactional data.

**Figure 3:**
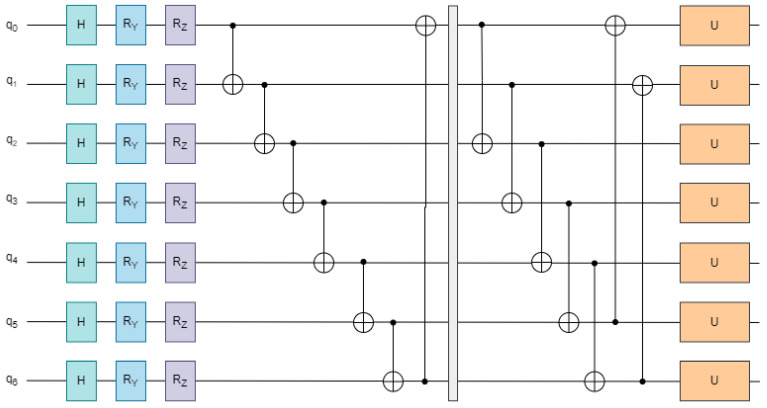
Structure of the quantum circuit for parameterized operations.

To enhance the expressive capacity of the PQC, a multi-layer stacking strategy is employed, as illustrated in Figure 3. Each layer consists of three sequential subcomponents: local entanglement, parameterized single-qubit rotation, and global entanglement. The complete unitary transformation across *L* stacked layers is formally defined as:

(7) $$U_{\mathrm{total}}=\prod_{l=1}^{L}\left[U_{\mathrm{ent}}^{\left(2\right)}\cdot U_{3}^{\left(l\right)}\cdot U_{\mathrm{ent}}^{\left(1\right)}\right]$$

In this expression, $U_{\mathrm{ent}}^{\left(1\right)}$ and $U_{\mathrm{ent}}^{\left(2\right)}$ represent the local and global entanglement operations, respectively, while $U_{3}^{\left(l\right)}$ denotes the parameterized rotation gates applied at the *l*-th layer. This hierarchical composition enables deeper quantum transformations and improves the circuit’s ability to capture complex feature dependencies and behavioral irregularities in transaction data.

#### 3.1.4. Quantum State Measurement and Output

Following quantum feature encoding and circuit evolution, the resulting high-dimensional quantum state must be translated into a classical representation for further processing by conventional deep learning components. To achieve this integration, the model performs standard Z-basis (Pauli-Z) measurements at the output of the VQCs. These measurements provide real-valued outputs that are both interpretable and compatible with classical architectures. Specifically, each qubit is measured using the following expectation value formulation:

(8) $$z_{i}=\langle \psi |Z_{i}|\psi \rangle ,\;i\in \left\{0,1,\ldots ,N-1\right\}$$

In this expression, |ψ〉∈H denotes the final quantum state vector within the $2^{N}$-dimensional Hilbert space H, and $Z_{i}$ is the Pauli-Z operator applied to the *i*-th qubit. The resulting scalar $z_{i}\in \left[-1,1\right]$ represents the expected outcome of the measurement, forming a vectorized classical representation that serves as input to subsequent layers of the hybrid model.

**Figure 4:**
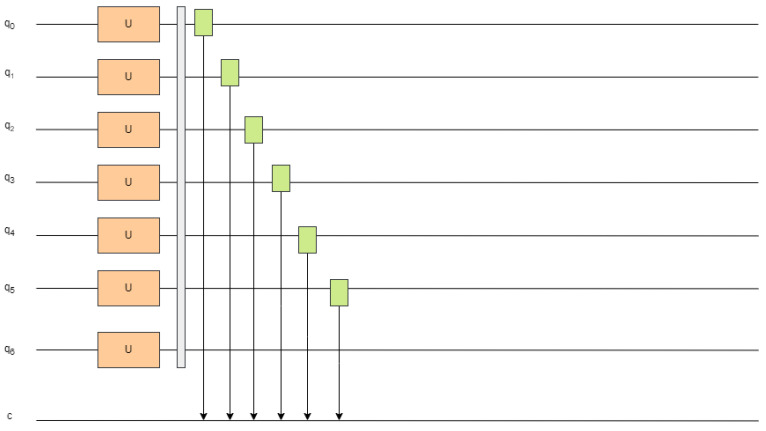
Schematic of quantum measurement and output generation.

As shown in Figure 4, the outcomes of all quantum measurements are aggregated into a fixed-length vector, forming a compact classical representation of the quantum-encoded input. This vector is fully compatible with the input format required by the subsequent classical deep learning components. It encapsulates the transformed and evolved quantum representation of the original transactional features and is used as the input at each time step within the downstream hybrid sequential model.

### 3.2. Hybrid Quantum Recurrent Neural Network Model

Financial transaction data are inherently nonlinear, temporally dependent, and behaviorally dynamic, which presents significant challenges for quantum encoders to represent effectively using a single static embedding.

To address these challenges, this study introduces HQRNN-FD, a hybrid quantum–classical architecture that combines the nonlinear feature mapping capability of VQCs with the temporal sequence modeling strengths of RNNs. In addition, a self-attention mechanism is incorporated to dynamically emphasize time steps that exhibit irregular or suspicious patterns within transaction sequences [21,32].

**Figure 5:**
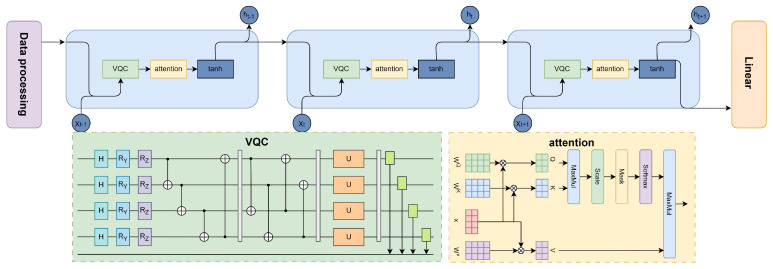
Architecture diagram of the hybrid quantum recurrent neural network.

As illustrated in Figure 5, the overall architecture of HQRNN-FD comprises three key components: a quantum encoding module that enhances nonlinear representational capacity, a recurrent path that captures sequential dependencies, and a self-attention layer that adaptively focuses on behaviorally significant temporal points most indicative of fraudulent activity.

Fraudulent behaviors are typically rare and manifest as subtle anomalies within a large volume of normal transactions, making them difficult to detect based solely on raw features. To address this issue, each transaction vector $x^{\left(t\right)}$ in the preprocessed sequence $X=\{x^{\left(1\right)},\ldots ,x^{\left(T\right)}\}$ is processed through a VQC, generating a corresponding measurement vector $z^{\left(t\right)}\in R^{N}$ that serves as a quantum-enhanced embedding of the original features.

Since fraudulent activity may arise from small deviations or inconsistent behavioral patterns distributed over time, each quantum-encoded vector $z^{\left(t\right)}$ is subsequently passed into an RNN unit. In this design, the RNN directly consumes quantum-enhanced feature vectors generated by the VQC at each time step, rather than raw transaction inputs. Through the dual-angle encoding and entanglement process, these embeddings already incorporate nonlinear, high-dimensional feature interactions, providing the recurrent layer with a richer and more discriminative representational basis. This quantum preprocessing embeds complex inter-feature relationships into a shared encoding space that remains consistent across time steps, thereby producing time-consistent feature representations that facilitate the RNN’s ability to recognize recurring and evolving patterns within the sequence. As a result, the RNN can capture long-range temporal dependencies among quantum-augmented features, enabling the detection of subtle, temporally correlated anomalies that might otherwise remain indistinguishable in the original feature space.

This enables the model to capture temporal dependencies through the recursive update rule:

(9) $$h^{\left(t\right)}=\mathrm{RNN}\left(z^{\left(t\right)},h^{\left(t-1\right)}\right)$$

RNNs are well suited for modeling sequential data, as they effectively capture temporal continuity and preserve historical context. However, they may have difficulty assigning appropriate importance to individual time steps, which can result in diminished sensitivity to critical anomalies. To address this limitation, a self-attention mechanism is incorporated to perform a weighted aggregation over the hidden states $h^{\left(t\right)}$, thereby enabling the model to focus selectively on informative temporal patterns.

**Figure 6:**
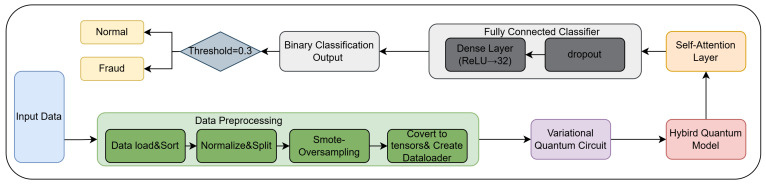
Workflow of the HQRNN-FD model.

The complete HQRNN-FD workflow for financial fraud detection is illustrated in Figure 6. This end-to-end architecture includes data preprocessing, quantum feature encoding, temporal modeling with attention, and final classification. The pipeline begins with input normalization and class balancing using SMOTE [33], followed by quantum representation learning through VQCs. The encoded features are then processed by a discriminative layer consisting of self-attention and fully connected components. The final prediction is produced by a threshold-based binary classifier, which identifies anomalous transaction behaviors indicative of potential fraud.

## 4. Data

### 4.1. Data Source

This study employs the Fraudulent Transaction Detection dataset, which contains 1,754,155 financial transaction records spanning various time periods and includes both legitimate and fraudulent activities. A summary of the dataset’s original feature set is provided in Table 1, comprising attributes such as transaction time, transaction amount, terminal identifier, customer identifier, and a binary label variable (TX_FRAUD) that indicates whether a transaction is classified as fraudulent.

To investigate seasonal trends in transaction behavior, a month-wise statistical analysis of the dataset was conducted. Table 2 presents the distribution of legitimate and fraudulent transactions for each month. The analysis reveals considerable variation in transaction volume over time, with a notably low number of samples recorded in July. This imbalance may limit the model’s capacity to accurately capture fraud-specific patterns occurring during that period.

### 4.2. Data Preprocessing

Financial transaction data are often characterized by high dimensionality, pronounced class imbalance, and strong temporal dependencies. If left unaddressed, these properties can result in model overfitting to majority-class (normal) samples and inadequate representation of minority-class (fraudulent) instances, thereby impairing detection performance.

**Figure 7:**
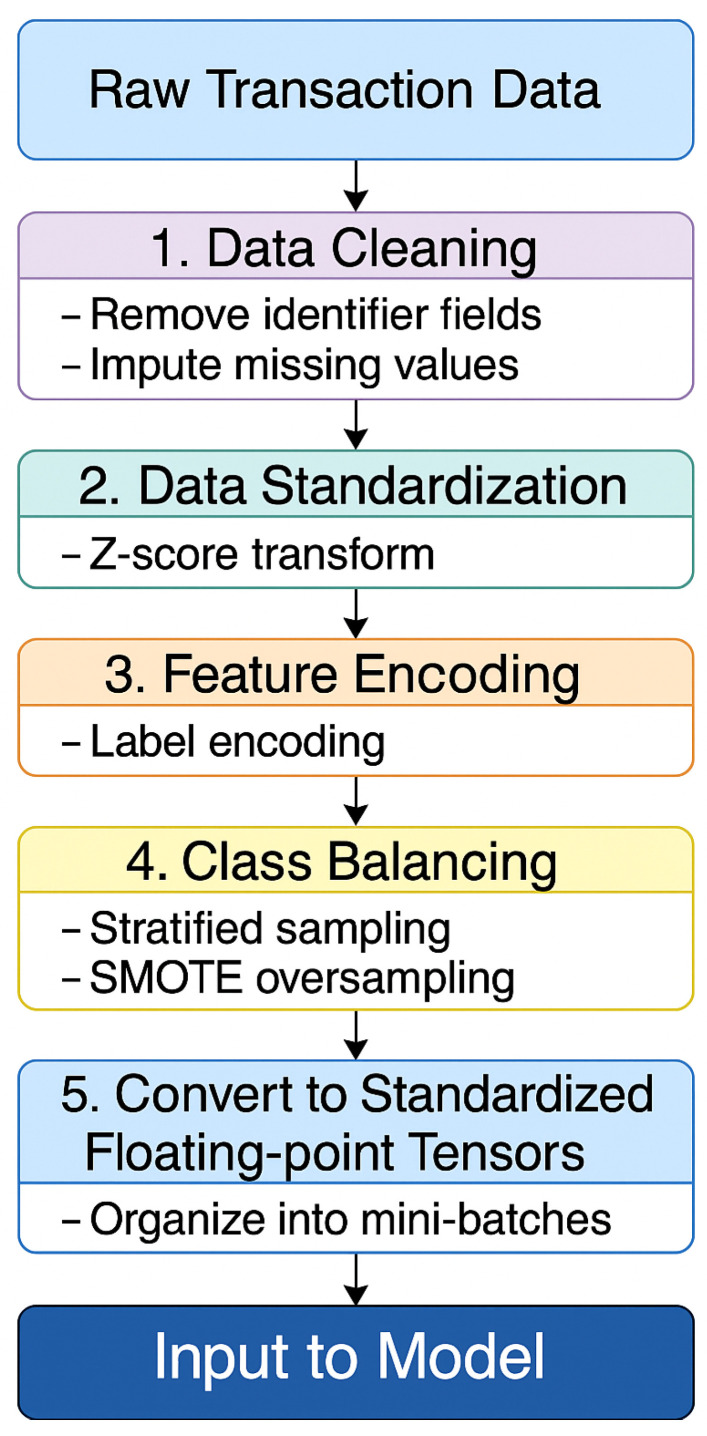
Diagram of the data preprocessing and feature encoding workflow.

To counter these challenges, a structured preprocessing pipeline is implemented. This pipeline includes data cleaning, feature encoding, class balancing through the SMOTE, and construction of tensor-based input representations. The overall workflow is depicted in Figure 7.

#### 4.2.1. Preprocessing Steps

The original dataset included irrelevant or non-informative fields, such as customer names and account numbers. These attributes were consistently removed during preprocessing to reduce data redundancy.

To manage missing values, time series interpolation was applied according to the type of variable, ensuring continuity and completeness in the transaction records.

Numerical features were standardized using Z-score normalization, which transformed them into distributions with zero mean and unit variance. This step supported stable training and improved convergence of the model.

For categorical variables, such as transaction types and terminal categories, label encoding was employed. This method was preferred over one-hot encoding, as it avoided high-dimensional sparsity and ensured compatibility with the sequence modeling and quantum feature extraction components.

#### 4.2.2. Handling Class Imbalance

The original dataset presents a significant class imbalance, with fraudulent transactions accounting for only a small fraction of the total records. Training directly on such imbalanced data can cause the model to become biased toward the majority class, leading to poor sensitivity in detecting fraudulent activity.

To address this issue, the dataset is divided into training and test sets using an 80% to 20% split, resulting in 1,403,325 training samples and 350,830 test samples. Within the training set, fraudulent transactions constitute only 13.5% of the total, highlighting the imbalance.

To improve class distribution, the SMOTE is applied to generate additional synthetic samples for the minority class. This enhances the model’s generalization capacity by expanding the representation of fraudulent patterns [20]. The generation of a synthetic instance is defined by the following equation:

(10) $$x_{\mathrm{new}}=x_{i}+\lambda \left(x_{ij}-x_{i}\right),\;\lambda ∼U\left(0,1\right)$$

In this formulation, $x_{i}$ is a minority class sample and $x_{ij}$ is one of its *k*-nearest neighbors within the same class. The interpolation coefficient λ is drawn from a uniform distribution U(0,1), allowing the new sample $x_{\mathrm{new}}$ to be generated along the line segment connecting $x_{i}$ and $x_{ij}$. This process enriches the minority class feature space and improves the model’s ability to detect fraudulent transactions.

**Figure 8:**
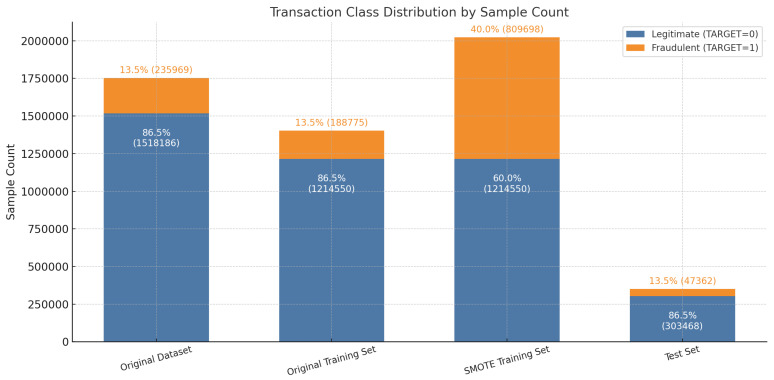
Class distribution comparison before and after applying SMOTE.

As shown in Figure 8, applying the SMOTE technique expanded the training set to 2,024,249 samples, increasing the fraud class proportion to 40% and resulting in an approximate 6:4 class balance. The test set maintained its original distribution, with fraudulent transactions comprising around 13.5%, ensuring that model evaluation remains aligned with real-world operational conditions.

## 5. Experiment

This section outlines the experimental framework, including the setup and analysis of results. The experiments are designed to investigate the following research questions:

- How effectively does the proposed HQRNN-FD model perform in financial fraud detection tasks?
- How does HQRNN-FD compare to leading classical and quantum models when handling imbalanced classification problems?
- To what extent does the model demonstrate robustness to quantum noise and scalability across different qubit configurations?

### 5.1. Description of the Experiments

#### 5.1.1. Experiment Setting

To thoroughly assess the effectiveness of the proposed HQRNN-FD model, a comprehensive set of comparative experiments is conducted, incorporating both conventional deep learning approaches and quantum-enhanced models [32,34]. The experimental design covers various aspects, including feature extraction techniques, recurrent structure modeling, self-attention mechanism integration, and quantum–classical hybrid frameworks.

**Figure 9:**
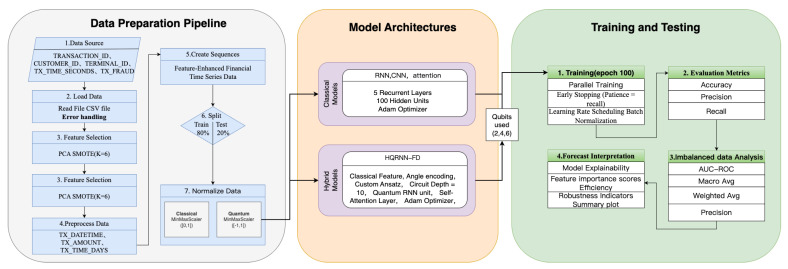
Architecture and workflow design of the financial fraud detection experiment.

The overall experimental process is divided into four stages: data preprocessing, model development, training strategy deployment, and performance evaluation. The design rationale and procedural flow are illustrated in Figure 9.

The proposed HQRNN-FD model utilizes shallow VQCs and evaluates configurations with 2, 4, and 6 qubits, keeping the circuit depth within the practical constraints of current NISQ hardware. Quantum simulations are performed using the PennyLane framework, which allows seamless transitions between different qubit settings. Model training is carried out using the Adam optimizer, with early stopping based on validation performance to prevent overfitting. A comprehensive overview of the experimental settings is provided in Table 3.

#### 5.1.2. Experiment Metrics

Financial fraud detection is a representative imbalanced classification problem, marked by complex data distributions and rare anomalous instances. To assess model effectiveness under these conditions, four key evaluation metrics derived from the confusion matrix are utilized, as summarized in Table 4.

Based on the aforementioned definitions, the primary evaluation metrics used to assess the model’s effectiveness in detecting financial fraud are outlined as follows:

- Accuracy: Assesses the overall correctness of classifications across both classes:
  (11) $$\mathrm{Accuracy}=\frac{TP+TN}{TP+TN+FP+FN}$$
- Precision: Indicates the ratio of true positive fraud predictions to the total number of transactions predicted as fraudulent:
  (12) $$\mathrm{Precision}=\frac{TP}{TP+FP}$$
- Recall: Reflects the model’s effectiveness in identifying true fraudulent transactions among all actual fraud cases:
  (13) $$\mathrm{Recall}=\frac{TP}{TP+FN}$$
- F1-score: The harmonic mean of precision and recall, assessing the balance between them:
  (14) $$\text{F1-score}=2\times \frac{\mathrm{Precision}\times \mathrm{Recall}}{\mathrm{Precision}+\mathrm{Recall}}$$
- Robustness to Quantum Noise: Gaussian quantum noise with standard deviations of 0.01, 0.05, and 0.1 is introduced into the test set. Variations in Precision, Recall, F1-score, and Accuracy are measured to assess the model’s resistance to noise-induced degradation.

### 5.2. Baseline Models

To ensure fair and reproducible comparisons, all baseline models were implemented with a consistent input dimensionality of 6 features per transaction (after preprocessing) and with comparable network capacities. Each model receives an input feature vector of dimension 6 after preprocessing, ensuring that the quantum and classical models are compared on the same input basis. The selected hyperparameters strike a balance between providing sufficient model capacity to capture complex patterns and preventing overfitting, consistent with prior studies on fraud detection and quantum–classical hybrid architectures.

- Convolutional Neural Network (CNN): A single one-dimensional convolutional layer with 32 filters (kernel size = 3), followed by a pooling layer and a fully connected output layer. This architecture effectively captures local temporal patterns in transaction sequences, making it suitable for detecting short-term anomalies, but it lacks the ability to model long-range sequential dependencies.
- Recurrent Neural Network (RNN): A single recurrent layer with 64 hidden units and tanh activation, followed by a dense output layer. RNNs can model sequential dependencies and temporal evolution in transaction data, offering stronger context modeling than CNNs, though they are susceptible to vanishing gradients and have limited long-range memory.
- Quantum Neural Network (QNN): A two-layer variational quantum circuit (VQC), where each layer consists of parameterized rotation gates (Ry and Rz) and entanglement operations implemented as a ring topology of nearest-neighbor CNOTs to ensure scalable inter-qubit correlations. The number of qubits *N* matches the input feature dimension (N=6). A classical dense layer follows after quantum expectation values are measured. While effective for nonlinear transformation in low-dimensional feature spaces, QNNs lack temporal modeling capacity.
- Quantum Recurrent Neural Network (QRNN): A two-layer VQC (6 qubits, ring-topology nearest-neighbor CNOT entanglement) for quantum encoding, followed by a classical RNN layer with 64 hidden units for sequential modeling. This hybrid architecture captures both high-dimensional quantum features and dynamic behavioral patterns over time, offering stronger temporal modeling than QNN alone.
- Hybrid Quantum Recurrent Neural Network (HQRNN): A baseline hybrid model connecting a fixed-topology VQC (6 qubits) to a shallow single-layer RNN with 64 hidden units. Due to structural decoupling—where the RNN does not adapt to the quantum encoding—and limited depth, it has reduced capability in detecting subtle or long-range fraud patterns.
- HQRNN-FD (Proposed Model): An enhanced hybrid quantum–classical architecture that addresses HQRNN’s limitations by aligning VQC depth with the recurrent architecture. The VQC uses 6 qubits to match the input feature dimension, while the RNN component comprises multiple recurrent blocks adaptively configured to the quantum topology. A self-attention module is applied to focus on salient transaction segments, improving discrimination of minority-class fraudulent behavior.

For all RNN-, QRNN-, HQRNN-, CNN-, and QNN-based models, Binary Cross-Entropy loss was used with a learning rate of 0.001 and 64 training epochs. For RNN-family models (RNN, QRNN, HQRNN), the hidden unit size was fixed at 64, with a single recurrent layer, tanh activation, and a linear prediction output layer. Both QRNN and HQRNN employ a two-layer VQC with 6 qubits and ring-topology entanglement (nearest-neighbor CNOTs) before the recurrent layer, consistent with their architectural descriptions in Section 5.2. For non-recurrent baselines, two widely adopted neural architectures were used: CNN and QNN. The CNN consists of a single one-dimensional convolutional layer with 32 filters (kernel size = 3), followed by pooling and a fully connected output layer. The QNN follows the structure in [34], using 6 input qubits to match the data dimensionality. These unified hyperparameter and training settings ensure strict fairness in the comparison across all baseline models.

### 5.3. Experimental Results

Comprehensive Performance Comparison: Financial fraud detection involves a pronounced class imbalance, with legitimate transactions making up for the vast majority and fraudulent transactions compromising less than 5% of the data. The key challenge is to improve the model’s capacity to accurately detect the minority class of high-risk fraudulent instances while minimizing false positives (FPs) among legitimate transactions, thereby ensuring dependable and efficient detection performance.

#### 5.3.1. Confusion Matrix Analysis

Variation in classification error distributions between normal and fraudulent transactions is clearly observed across different models (Table 5). For instance, the CNN model recorded 19,711 FP, markedly higher than those produced by the RNN (13,597) and HQRNN-FD (8110). Elevated FP rates can result in excessive rejection of legitimate transactions, leading to increased user dissatisfaction and higher manual verification costs.

In contrast, HQRNN-FD achieves a lower FP count while maintaining a manageable number of false negatives (FN) for fraudulent cases at 1891. Although the original HQRNN demonstrates strong recall (FN = 1777), its higher FP count indicates reduced precision in detecting normal transactions.

While QNN and QRNN show moderate effectiveness in minimizing FN (1800 and 2446, respectively), they will exhibit relatively high FP values (15,450 for QNN and 11,399 for QRNN). In particular, QRNN prioritizes recall at the expense of precision.

**Figure 10:**
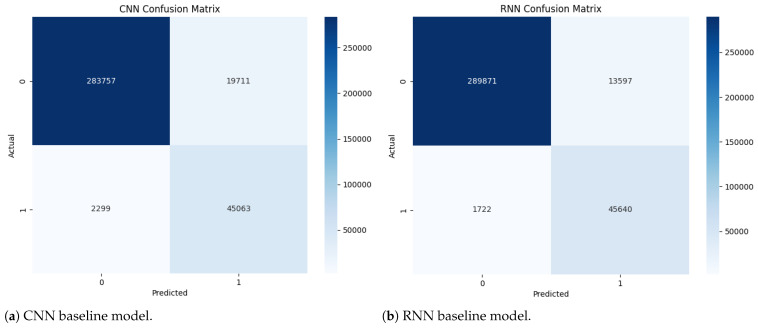

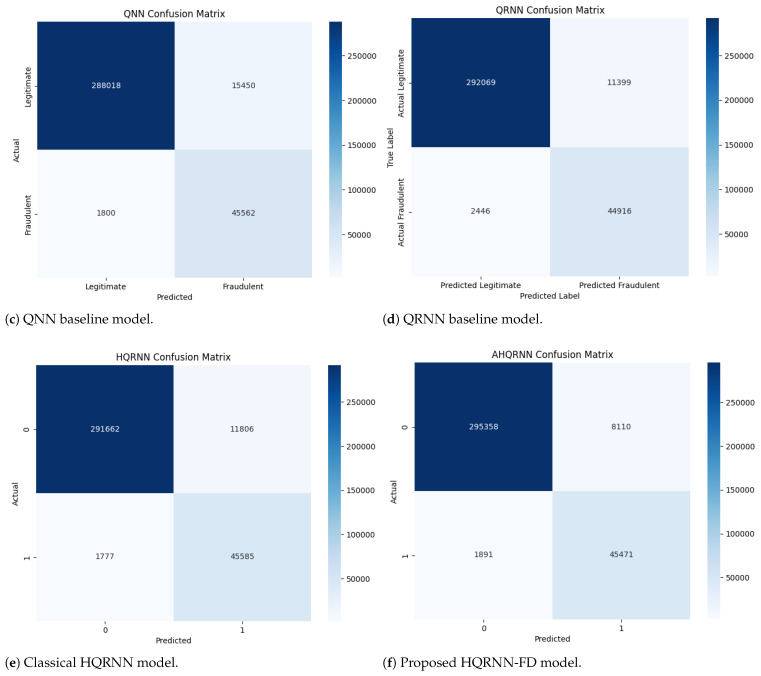
Architectural comparison of fraud detection models across classical, quantum, and hybrid quantum–classical approaches. Subfigures (**a**,**b**) show classical deep learning models: CNN and RNN. Subfigures (**c**,**d**) illustrate quantum-only frameworks: QNN and QRNN. Subfigures (**e**,**f**) present hybrid designs: the classical HQRNN and the proposed HQRNN-FD. All models are evaluated based on their detection accuracy, robustness, and generalization capabilities under imbalanced transaction conditions.

Overall, HQRNN-FD consistently achieves the lowest FP and FN rates, effectively minimizing false alerts while preserving high detection sensitivity (Figure 10).

#### 5.3.2. Model Metrics Analysis

In terms of classification metrics, HQRNN-FD attained the highest F1-score of 0.9009 for the fraud class (Class 1), surpassing all baseline models. Compared with the original HQRNN, it showed a notable improvement in precision, from 0.7943 to 0.8486, and an approximate 3% increase in F1-score, highlighting the effectiveness of integrating quantum encoding with attention-based mechanisms.

Moreover, HQRNN-FD achieved superior performance in both macro and weighted average F1-scores, recording values of 0.9421 and 0.9722, respectively, which reflects strong performance across all classes. In comparison, conventional models, such as CNN and DNN, showed reasonable results on normal transactions but performed poorly on fraudulent cases, with F1-scores of 0.8037 and 0.8294, respectively, indicating reduced accuracy and a higher misclassification risk.

While QNN and QRNN leverage quantum-based components, their effectiveness is limited by shallow circuit architectures, resulting in overall F1-scores of 0.8408 and 0.8665, both of which remain lower than that of HQRNN-FD.

#### 5.3.3. Noise Resistance Analysis

Quantum circuits are inherently susceptible to various noise types, such as depolarizing, bit-flip, and phase-flip noise, particularly when deployed on current NISQ devices. This section investigates the robustness of the HQRNN-FD model relative to other quantum baselines (HQRNN, QNN, and QRNN) under three representative noise models. Each model was evaluated at noise intensities of 0 (ideal), 0.01, 0.05, and 0.10, with the results summarized in Table 6.

Depolarizing Noise Impact: Depolarizing noise introduces random qubit decoherence over the Bloch sphere. The Kraus operator for depolarizing noise is expressed as:

(15) $$E_{\mathrm{depolarizing}}\left(\rho \right)=\left(1-p\right)\cdot \rho +\frac{p}{3}\cdot \left(X\rho X^{†}+Y\rho Y^{†}+Z\rho Z^{†}\right),$$

where *p* denotes the depolarization probability, and *X*, *Y*, and *Z* represent the Pauli operators. This type of noise randomly applies one of the Pauli operators to a qubit with equal probability. Under low noise levels (0.01, 0.05), HQRNN-FD shows minimal performance degradation, with its F1-score slightly decreasing from 0.9009 to 0.8914 and 0.8906, respectively, while maintaining an accuracy above 96.8%. Even at higher noise intensity (0.10), the model preserves a competitive F1-score of 0.8455 and an accuracy of 95.28%, outperforming most baseline methods and confirming robustness to quantum decoherence.

By contrast, the QNN model exhibits relatively stable performance but maintains lower precision, with a modest decline in F1-score from 0.8408 to 0.8356. However, the QRNN model demonstrates higher sensitivity to depolarizing noise, as evidenced by a substantial drop in F1-score from 0.9768 to 0.7635, suggesting a significant reduction in its generalization capability under noisy conditions.

Impact of Bit-Flip Noise: Bit-flip noise results in the inversion of quantum states between |0〉 and |1〉, which can significantly impair classification performance. It is represented by the following Kraus operator formulation:

(16) $$E_{\text{bit-flip}}\left(\rho \right)=\left(1-p\right)\cdot \rho +p\cdot X\rho X^{†},$$

where *p* represents the bit-flip probability and *X* corresponds to the Pauli-X operator. Under high noise conditions (0.10), the F1-score of HQRNN-FD drops to 0.8214, yet it still surpasses both HQRNN (0.8213) and QNN (0.5757). At a moderate noise level (0.05), HQRNN-FD maintains a consistent F1-score of 0.8590, demonstrating enhanced robustness to noise.

By contrast, QRNN exhibits pronounced sensitivity to bit-flip noise, with its F1-score falling sharply to 0.5754 and accuracy decreasing to 83.97% at a noise level of 0.10, indicating limited robustness against such perturbations.

Impact of Phase-Flip Noise: Phase-flip noise alters the phase of qubits and has a particularly adverse effect on models utilizing angle-based encoding. It is characterized by the following Kraus operator expression:

(17) $$E_{\text{phase-flip}}\left(\rho \right)=\left(1-p\right)\cdot \rho +p\cdot Z\rho Z^{†},$$

where *p* denotes the phase-flip probability and *Z* is the Pauli-Z operator. HQRNN-FD exhibits high resilience to phase perturbations, maintaining F1-scores between 0.8943 and 0.8913 across noise levels ranging from 0.01 to 0.10. Its classification accuracy remains consistently above 96.8%, surpassing the performance of HQRNN, QNN, and QRNN.

Notably, both QNN and QRNN experience considerable performance degradation in the presence of phase-flip noise. Specifically, QNN’s F1-score declines to 0.5919 under high noise intensity (0.10), indicating limited robustness to phase disturbances.

#### 5.3.4. Scalability of the HQRNN-FD Model

In hybrid QNN architectures, the number of qubits plays a critical role in determining the representational power and encoding depth of quantum components. However, increasing qubit count also leads to higher computational complexity and poses additional challenges for parameter optimization.

**Figure 11:**
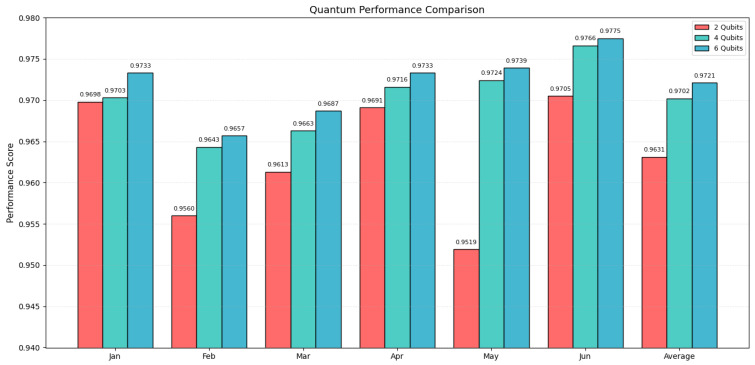
Monthly accuracy under different qubit configurations.

To assess scalability, the HQRNN-FD model is used as a representative case, with classification performance evaluated under 2-, 4-, and 6-qubit configurations. Accuracy is measured over six monthly intervals from January to June 2023. The corresponding results are presented in Table 7 and illustrated in Figure 11.

The overall results suggest a consistent improvement in classification accuracy as the number of qubits increases. Notably, the 6-qubit configuration enabled HQRNN-FD to achieve the highest accuracy in 5 out of 6 monthly evaluations, with an average accuracy of 0.9721, surpassing the 4-qubit and 2-qubit configurations, which attained 0.9702 and 0.9631, respectively. This performance advantage is particularly evident in February and May, months marked by greater data imbalance, demonstrating the enhanced stability and adaptability of the 6-qubit configuration under challenging conditions.

As shown in Figure 11, the monthly accuracy comparison across different qubit configurations highlights notable performance differences. The 2-qubit model consistently yields lower accuracy, particularly in February (0.9560) and May (0.9519), suggesting a representational limitation in capturing complex transaction dynamics. Conversely, the 4-qubit configuration strikes a practical balance between accuracy and quantum resource usage. Its performance in April and June closely approximates that of the 6-qubit model, indicating its stability for scenarios where computational resources are limited.

Regarding average accuracy, increasing the number of qubits positively influences model performance, though not in a strictly linear fashion. The 4-qubit configuration demonstrates reliable modeling capability, whereas the 6-qubit setup yields further improvements in accuracy, making it more suitable for high-precision fraud detection applications within financial risk management contexts.

#### 5.3.5. Ablation Study Analysis

To quantitatively evaluate the contributions of individual components within the HQRNN-FD framework and examine the efficacy of preprocessing, a systematic ablation study was conducted. This involved selectively disabling key modules—namely, variational quantum circuits (VQCs), self-attention mechanisms, and recurrent neural networks (RNNs)—to create controlled model variants. Each configuration was assessed using an identical test dataset, with results detailed in Table 8.

A direct comparison between HQRNN and HQRNN-FD demonstrates that incorporating the attention mechanism enhances the Class 1 F1-score from 0.8703 to 0.9009, indicating improved identification of salient behavioral features within transactional sequences and strengthening anomaly detection performance. Precision also improved from 0.7943 to 0.8486, reflecting an enhanced reduction in false positives (FPs). These outcomes emphasize the synergy between quantum feature extraction and attention mechanisms in sequential fraud pattern recognition.

By contrast, the HQRNN-FD variant without RNNs retained quantum feature encoding but showed limited temporal modeling capabilities due to the absence of recurrent processing. This resulted in a decrease in F1-score from 0.9009 to 0.8665, indicating that quantum encoding alone is insufficient to capture complex temporal dependencies and fraud-related patterns. The RNN component is essential for processing sequences of transaction features across time, capturing long-range correlations and temporal evolution of user behavior. In particular, the RNN with attention enables the model to focus on informative segments of transaction histories (e.g., irregular intervals of high-risk activity), thus providing context-awareness that purely quantum or static models, such as QNNs, lack.

Meanwhile, the HQRNN-FD configuration without VQCs achieved consistently high recall values (all exceeding 0.96) but suffered from reduced precision and F1-scores, reflecting a tendency to misclassify normal transactions as fraudulent (i.e., over-detection). This further highlights the complementary role of the quantum encoder; by enhancing the representational richness of each transaction’s feature vector, it provides more informative input to the RNN, improving discrimination between subtle classes.

Taken together, these findings validate that the integration of quantum encoding with temporal modeling substantially enhances the robustness and effectiveness of the framework, particularly in scenarios characterized by class imbalance. The superior performance of HQRNN-FD arises from this hybrid synergy—quantum circuits extract expressive nonlinear features at each time step, while RNNs learn the dynamics of transaction evolution. Ablation analysis confirms that removing either component undermines performance, and that the RNN is indispensable for effective sequence modeling.

Preprocessing Impact Assessment: The removal of the preprocessing pipeline—particularly SMOTE oversampling—consistently led to performance degradation across all model configurations, most notably in precision and F1-score. For example, HQRNN-FD’s precision dropped from 0.8486 to 0.7797, and its F1-score declined from 0.9009 to 0.8694, accompanied by a modest accuracy decrease of 0.52% (from 97.15% to 96.66%). These results emphasize the essential role of preprocessing in strengthening model robustness and enhancing the detection of minority-class fraudulent instances.

The adverse impact was more severe in classical variants. HQRNN-FD without attention and VQCs, as well as the version without VQCs alone, experienced significant F1-score reductions to 0.8020 and 0.8320, respectively—underscoring the vulnerability of deep learning models to class imbalance. Notably, the RNN-removed variant (HQRNN-FD w/o RNNs) showed an increase in recall (0.9656) in the absence of preprocessing; however, this was offset by a substantial drop in F1-score, suggesting that higher recall came at the cost of more false positives, ultimately compromising the model’s reliability and interpretability. This performance drop was consistent across precision, recall, and accuracy metrics, reinforcing the robustness of the RNN’s contribution across evaluation criteria.

## 6. Conclusions

This study tackles the challenges of modeling complex nonlinear transaction patterns and class imbalance in financial fraud detection by introducing a hybrid deep neural network, HQRNN-FD, which integrates quantum-based feature representation with temporal sequence modeling. The architecture combines VQCs, efficient recurrent units, and self-attention mechanisms to enhance the model’s capacity for precise pattern recognition and sensitivity to subtle fraudulent signals.

Experimental findings demonstrate that HQRNN-FD surpasses several classical and quantum benchmark models in key performance metrics—including precision, recall, and F1-score—with particular effectiveness in identifying fraudulent (Class 1) instances. Furthermore, evaluations under various quantum noise models (e.g., depolarization, bit-flip, and phase errors) confirm the model’s robustness, while qubit scalability tests affirm its performance gains with increasing quantum resources.

The superior performance of HQRNN-FD is driven by the synergistic integration of quantum encoding, temporal memory via RNNs, and attention-based focus. The VQC module excels at mapping transaction attributes into expressive, nonlinear quantum feature spaces, while the RNN captures temporal dependencies and long-range behavioral patterns, and the self-attention mechanism selectively emphasizes time steps that exhibit anomalous behavior. This synergy is essential for accurately identifying minority-class fraudulent behavior in imbalanced financial datasets. Compared to purely classical or purely quantum counterparts, the hybrid design equips HQRNN-FD with both the expressivity of quantum transformations and the temporal reasoning capacity of recurrent modeling, augmented by attention’s ability to localize critical behavioral cues.

Ablation results (Section 5.3.5) confirm the critical role of the RNN, with measurable performance degradation observed when it is removed. This complementary division of labor—feature enrichment in the quantum domain, sequence learning in the recurrent domain, and focused pattern extraction via attention—enables the hybrid model to detect both fine-grained transactional anomalies and their temporal evolution, capabilities that none of the individual modules could achieve as effectively in isolation.

In summary, HQRNN-FD consistently achieves accurate detection of minority-class fraud cases while maintaining a low false positive rate, underscoring the potential of quantum–classical hybrid architectures in financial risk management. Future directions include exploring deployment on quantum hardware and integrating heterogeneous financial datasets to support broader real-world applications of QML in high-stakes financial environments. It should also be acknowledged that the present study is limited to structured transaction data. In real-world fraud detection, contextual, behavioral, and social signals may also provide valuable cues. Future research will extend HQRNN-FD to multi-modal settings by incorporating diverse data sources—such as customer interaction logs, device metadata, and social network information—to further enhance detection robustness and applicability.

## Figures and Tables

**Table 1:** Description of attributes in the original financial transaction dataset.

Attribute Name	Description
TRANSACTION_ID	Unique identifier for each transaction
TX_DATETIME	Date and time when the transaction occurred
CUSTOMER_ID	Unique identifier for each customer
TERMINAL_ID	Terminal through which the transaction took place
TX_AMOUNT	Amount of the transaction
TX_TIME_SECONDS	Seconds elapsed before the transaction
TX_TIME_DAYS	Days elapsed before the transaction
TX_FRAUD	Label: 0 (legitimate), 1 (fraudulent)

**Table 2:** Distribution of legitimate and fraudulent transactions by month.

Month	Legitimate Transactions	Fraudulent Transactions	Total Transactions
January	258,375	39,265	297,640
February	232,475	36,195	268,670
March	256,531	40,165	296,696
April	248,685	38,679	287,364
May	256,655	40,218	296,873
June	248,986	38,854	287,840
July	16,477	2595	19,072

**Table 3:** Parameter settings used in HQRNN-FD model experiments.

Parameter	Details
Dataset	Public credit card fraud detection dataset
Data split	80% training set, 20% test set
Baseline models	CNN, RNN, QRNN [32], QNN [34]
Number of qubits	2, 4, 6
Optimization algorithm	Adam optimizer with learning rate scheduling
Evaluation metrics	Accuracy, F1-score
Hyperparameters	Dropout: 0.2; Batch size: 256; Max epochs: 100; Early stopping enabled

**Table 4:** Confusion matrix for binary classification.

	Predicted Positive	Predicted Negative	Total
**Actual positive**	True positive (TP)	False negative (FN)	P
**Actual negative**	False positive (FP)	True negative (TN)	N

**Table 5:** Comparison of key performance metrics across different models.

Model	Precision	Recall	F1-Score	Model	Precision	Recall	F1-Score
HQRNN-FD				HQRNN			
Class 0	0.9936	0.9733	0.9834	Class 0	0.9939	0.9611	0.9772
Class 1	0.8486	0.9601	0.9009	Class 1	0.7943	0.9625	0.8703
Accuracy			0.9715	Accuracy			0.9613
Macro avg	0.9211	0.9667	0.9421	Macro avg	0.8941	0.9618	0.9238
Weighted avg	0.9741	0.9715	0.9722	Weighted avg	0.9670	0.9613	0.9628
QRNN [32]				QNN [34]			
Class 0	0.9917	0.9624	0.9768	Class 0	0.9938	0.9491	0.9709
Class 1	0.7976	0.9484	0.8665	Class 1	0.7468	0.9620	0.8408
Accuracy			0.9605	Accuracy			0.9508
Macro avg	0.8946	0.9554	0.9217	Macro avg	0.8703	0.9555	0.9059
Weighted avg	0.9655	0.9605	0.9619	Weighted avg	0.9604	0.9508	0.9534
CNN				RNN			
Class 0	0.9920	0.9350	0.9627	Class 0	0.9941	0.9552	0.9743
Class 1	0.6957	0.9515	0.8037	Class 1	0.7705	0.9636	0.8563
Accuracy			0.9373	Accuracy			0.9563
Macro avg	0.8438	0.9433	0.8832	Macro avg	0.8823	0.9594	0.9153
Weighted avg	0.9520	0.9373	0.9412	Weighted avg	0.9639	0.9563	0.9583

**Table 6:** Performance metrics of different models under quantum noise.

Model	Precision	Recall	F1-Score	Accuracy
HQRNN-FD	0.8486	0.9601	0.9009	0.9715
Depolarizing (0.01)	0.8312	0.9610	0.8914	0.9684
Depolarizing (0.05)	0.8307	0.9599	0.8906	0.9682
Depolarizing (0.10)	0.7573	0.9570	0.8455	0.9528
Bit-flip (0.01)	0.8225	0.9597	0.8858	0.9666
Bit-flip (0.05)	0.7846	0.9489	0.8590	0.9579
Bit-flip (0.10)	0.7389	0.9247	0.8214	0.9457
Phase-flip (0.01)	0.8310	0.9611	0.8913	0.9684
Phase-flip (0.05)	0.8335	0.9607	0.8926	0.9688
Phase-flip (0.10)	0.8367	0.9603	0.8943	0.9693
HQRNN	0.7943	0.9625	0.8703	0.9613
Depolarizing (0.01)	0.7619	0.9641	0.8512	0.9545
Depolarizing (0.05)	0.7618	0.9637	0.8510	0.9544
Depolarizing (0.10)	0.7592	0.9630	0.8491	0.9538
Bit-flip (0.01)	0.7620	0.9641	0.8512	0.9545
Bit-flip (0.05)	0.7536	0.9631	0.8456	0.9525
Bit-flip (0.10)	0.7199	0.9560	0.8213	0.9438
Phase-flip (0.01)	0.7619	0.9640	0.8511	0.9545
Phase-flip (0.05)	0.7541	0.9629	0.8458	0.9526
Phase-flip (0.10)	0.7214	0.9560	0.8223	0.9442
QNN [34]	0.7468	0.9620	0.8408	0.9508
Depolarizing (0.01)	0.7461	0.9621	0.8404	0.9507
Depolarizing (0.05)	0.7428	0.9623	0.8384	0.9499
Depolarizing (0.10)	0.7383	0.9625	0.8356	0.9489
Bit-flip (0.01)	0.7132	0.9358	0.8095	0.9405
Bit-flip (0.05)	0.5908	0.8416	0.6943	0.8999
Bit-flip (0.10)	0.4688	0.7455	0.5757	0.8516
Phase-flip (0.01)	0.6971	0.9588	0.8073	0.9382
Phase-flip (0.05)	0.5535	0.9447	0.6980	0.8896
Phase-flip (0.10)	0.4357	0.9226	0.5919	0.8283
QRNN [32]	0.7976	0.9484	0.8665	0.9605
Depolarizing (0.01)	0.7970	0.9477	0.8658	0.9604
Depolarizing (0.05)	0.7682	0.9242	0.8390	0.9521
Depolarizing (0.10)	0.6886	0.8566	0.7635	0.9284
Bit-flip (0.01)	0.7436	0.9316	0.8270	0.9474
Bit-flip (0.05)	0.5799	0.8702	0.6960	0.8974
Bit-flip (0.10)	0.4478	0.8048	0.5754	0.8397
Phase-flip (0.01)	0.7837	0.9390	0.8544	0.9568
Phase-flip (0.05)	0.7335	0.9072	0.8111	0.9430
Phase-flip (0.10)	0.6778	0.8691	0.7616	0.9265

**Table 7:** HQRNN-FD monthly accuracy under various qubit configurations.

Month	2 Qubits	4 Qubits	6 Qubits
January	0.9698	0.9703	0.9733
February	0.9560	0.9643	0.9657
March	0.9613	0.9663	0.9687
April	0.9691	0.9716	0.9733
May	0.9519	0.9724	0.9739
June	0.9705	0.9766	0.9775
Average	0.9631	0.9702	0.9721

**Table 8:** Comparative analysis of preprocessing impact on model performance.

Model	With Preprocessing				Without Preprocessing			
	Precision	Recall	F1-Score	Accuracy	Precision	Recall	F1-Score	Accuracy
HQRNN-FD	0.8486	0.9601	0.9009	0.9715	0.7797	0.9550	0.8694	0.9666
HQRNN-FD (w/o Attention)	0.7943	0.9625	0.8703	0.9613	0.7297	0.9500	0.8338	0.9517
HQRNN-FD (w/o RNNs)	0.7976	0.9484	0.8665	0.9605	0.7095	0.9656	0.8180	0.9420
HQRNN-FD (w/o Attention & VQCs)	0.7705	0.9636	0.8563	0.9563	0.7011	0.9340	0.8020	0.9372
HQRNN-FD (w/o VQCs)	0.8102	0.9650	0.8805	0.9650	0.7350	0.9450	0.8320	0.9500

## Data Availability

The codes and datasets used in this work are available from the corresponding author upon reasonable request.
